# Protective Effect of UP446 on Ligature-Induced Periodontitis in Beagle Dogs

**DOI:** 10.3390/dj7020033

**Published:** 2019-03-28

**Authors:** Mesfin Yimam, Lidia Brownell, Seon-Gil Do, Young-Chul Lee, Dong Seon Kim, Kangmoon Seo, Manbok Jeong, Seeun Kim, Qi Jia

**Affiliations:** 1 Unigen Inc., 2121 South State Street, Suite #400, Tacoma, WA 98405, USA; lbrownell@unigen.net (L.B.); jia@unigen.net (Q.J.); 2 Univera, Inc. 78, Achasan-ro, Seongdong-gu, Seoul 04789, Korea; sgildo@univera.com; 3 Naturetech, Inc. 29-8, Yongjeong-gil, Chopyeong-myeon, Jincheon-gun, Chungcheongbuk-do 27858, Korea; yclee@unigen.net; 4 Korea Institute of Oriental Medicine. 1672, Yuseong-daero, Yuseong-gu, Daejeon 34054, Korea; dskim@kiom.re.kr; 5 Department of Veterinary Clinics, College of Veterinary Medicine, Seoul National University. 1, Gwanak-ro, Gwanak-gu, Seoul 08826, Korea; kmseo@snu.ac.kr (K.S.); 9757044@hanmail.net (M.J.); seny27@hanmail.net (S.K.)

**Keywords:** periodontitis, periodontal disease, beagle dogs, *Scutellaria baicalensis*, *Acacia catechu*, UP446

## Abstract

Periodontal disease is an inflammatory disease of the gum caused by a formation of a plaque that triggers immune responses and inflammation leading to the destruction of tissues surrounding and supporting the teeth. Chronic usage of synthetic chemicals and antibiotics is limited by undesired adverse events to the host. A botanical composition (UP446), which consists primarily of bioflavonoids such as baicalin from roots of *Scutellaria baicalensis* and catechins from heartwoods of *Acacia catechu*, was evaluated for its effect on ligature-induced periodontal disease in beagle dogs. Disease model was induced in 20 male and female dogs. After a 12-week induction of periodontitis, animals were assigned to a placebo, positive control (doxycycline), and two treatment groups consisting of five animals each. The placebo group was only administrated to normal dog chow (25 g/kg/day). In the doxycycline treatment group, animals were fed a normal diet (25 g/kg/day) and doxycycline (5 mg/kg) was orally administrated every day. Treatment of UP446 was done by feeding the regular diet formulated with 0.1% and 0.2% of UP446 by weight. Clinical indices such as plaque index (PI), gingival index (GI), clinical attachment level (CAL), probing pocket depth (PPD), and bleeding on probing (BoP) were measured every two weeks for 12 weeks. UP446 administered to beagle dogs for 12 weeks at 0.1% and 0.2% resulted in statistically significant reductions in gingivitis, pocket depth, loss of attachment, and gum bleeding. UP446 could potentially be used alone or as an adjunct with other oral hygiene preparations for periodontal disease in both human and companion animals.

## 1. Introductions

Periodontal disease (PD) is an extremely prevalent inflammatory disease of the gum both in humans and canines [1,2,3]. It is characterized by progressive destruction of gingival soft tissue and alveolar bone caused by an invasive and persistent bacterial insult to the periodontium. The periodontium is the supporting structure of a tooth that encompasses the gingiva, alveolar bone, periodontal ligament, and cementum. Gingivitis is inflammation of the gum characterized by edematous appearance and erythema of the gingiva. If untreated it can progress to periodontitis leading to the damage of gum tissues and bone absorption. They are caused by a formation of a plaque, a pathogenic microbiota in the subgingival biofilm, which triggers innate and adaptive immune responses leading to the destruction of the tissues surrounding and supporting the teeth, with an eventual tooth loss [4]. Periodontal disease affects 20–50% of the global population [3]. It is the most prevalent disease in companion carnivores with prevalence estimates of between 44% and 64% [5], specifically with more than 80% for dogs aged two years or older [6]. This disease may also impact the incidence of diabetes; cardiovascular (atherosclerosis and bacterial endocarditis), kidney, rheumatologic, and respiratory diseases; and could also be a risk factor for premature childbirth [7]. Therapy modalities range from disease control and prevention (e.g., by plaque and calculus removal), non-surgical periodontal therapy (e.g., Scaling and closed root debridement), surgical therapy (e.g., mucogingival surgery and open curettage), use of antibiotics (e.g., doxycycline), and new therapies such as inducing periodontal regeneration [1].

Through the years, oral hygiene has been dependent on the use of synthetic chemical agents in preparations for mouth rinse and tooth paste in conjunction with antibiotics [8]. However, as a result of repeated exposure, oral microbes have developed resistance to antibiotics and synthetic chemicals reflected in the decreased clinical efficacy of oral hygiene products. In addition, synthetic substances also have several undesired adverse effects including vomiting, diarrhea, and teeth staining. Given these limitations, medicinal plants are now considered a new front as an alternative antimicrobial source to be incorporated into mouth rinses and toothpastes.

Inflammation and periodontal pathogens are the two primary risk factors for periodontal disease [1,2]. It is known that periodontitis is mainly a local inflammatory reaction initiated by plaque-associated bacteria in predisposed hosts. UP446 is a proprietary blend of *Scutellaria baicalensis* Georgi (Family: Lamiaceae) and *Acacia catechu* (Family: Mimosaceae), which were discovered through random screening of more than 1200 plant extracts using cyclooxygenase and lipoxygenase enzymatic assay. It has been previously shown to reduce production of eicosanoids and leukotrienes through dual inhibition of cyclooxygenase (COX) and lipoxygenase (LOX) enzymes. UP446 efficacies are the result of its active constituents primarily of baicalin and catechin. A different formulation of this composition is currently in use as a dietary supplement primarily for joint support. There are plenty of evidences that indicate the active components of the bioflavonoid composition UP446, baicalin and catechin, to possess activities (such as anti-inflammatory and antimicrobial) suggestive of their use in periodontal disease. Both baicalin and catechin have strong anti-inflammatory activities and have long been widely used as anti-inflammatory agents [9,10]. With a direct relevance to the current study, in a ligature and *Porphyromonas gingivalis* induced periodontitis model in rats, baicalin reduced alveolar bone loss, levels of HMGB1, TNF-α, and IL-1β significantly, in References [11,12]; in a similar model, baicalin prevented alveolar bone loss, and maintained high area fraction of collagen fiber through significant reductions in the expression of COX-2 and inducible nitric oxide synthase [13], as well as inhibition of expression of MMP-1 and MMP-9 [14]. Promoting the human periodontal ligament cell growth and differentiation [15], inhibiting IL-1 beta-induced synthesis of PGE2 and LTB4 and enhancing collagen synthesis in human gingival fibroblast [16], downregulating IL-6 and IL-8 expression in human oral keratinocytes [17], decreasing receptor activator of nuclear factor-κB ligand (RANKL) mRNA in cultured human periodontal ligament cells [18] are some of the significant activities of baicalin indicative of its usage in periodontal disease. Similarly, the other major active component of UP446, catechin has many pharmacological effects and beneficial properties, including anti-inflammatory, anti-oxidative, anticarcinogenic, and antimicrobial properties which have been reported to promote overall health [10]. Catechin administered at 200 mg/kg in ligature induced rat periodontitis model, reduced alveolar bone loss, down regulated IL-6, and TNF-alpha expression indicating its therapeutic effect on damaged periodontal tissue [19]. Epidemiologically, it has also been reported that regular intakes of green tea—a rich source of conjugated catechins—prevented the development and progression of chronic periodontitis and reduced the odds for tooth loss where it further signifies the potential usage of catechins in oral care [20].

Therefore, we hypothesized that UP446, a standardized bioflavonoid composition, which consists primarily of baicalin from roots of *Scutellaria baicalensis* Georgi and catechin from heartwoods of *Acacia catechu* Willd could act as anti-inflammatory agents to provide protection against periodontal disease, therefore proceeded to evaluate its clinical effects on ligature-induced periodontal disease in beagle dogs. Efficacy was compared against a positive control doxycycline. Doxycycline, a broad-spectrum antibiotic, belongs to a class of drugs known as tetracycline. It is commonly prescribed for bacterial tooth infection and known to prevent further bacterial growth

## 2. Materials and Methods

### 2.1. Preparation of UP446

Detailed method for preparation of the two standardized bioflavonoid extracts containing baicalin and catechin, from the roots of *S. baicalensis* and the heartwoods of *A. catechu*, respectively, were disclosed in a US patent [21]. Briefly, *S. baicalensis* root was extracted with hot water and then the bioflavonoids were crystallized from the aqueous solution with baicalin as the major component at a content not less than 75%. Catechin extract was obtained from recrystallization of an aqueous extract of the heartwoods of an *A. catechu*. Total catechin and epicatechin are no less than 65%. UP446, a proprietary blending of these extracts contains baicalin not less than 60% and catechin not less than 10% by weight in the final composition.

### 2.2. Study Design

The objective of this study was to determine the effect of UP446 on ligature-induced periodontal disease in beagle dogs. The study protocol was approved by Animal Studies Committee at Seoul National University, Seoul, Korea (Approval number: SNU-090219-2; Approval data: 19 February 2009). Six male and sixteen female dogs, 12 months of age, were studied. A sample size of five beagles per treatment group and four treatment groups were planned for this study. The experimental design included pretreatment and treatment phases. During the pretreatment phase, all dogs were maintained healthy gingival condition during 2 weeks after dental prophylaxis. Prophylactic procedures carried out by using the ultrasonic scaling for removal of dental calculus in gingival margin, polishing using pumice, and tooth brushing daily. The gingival health of each animal was measured by recording the presence or absence of gingival inflammation, plaque, and bleeding upon probing.

After the animals were brought to periodontal health, experimental periodontitis was induced for 12 weeks (Periodontal Disease Induction Phase). A shallow notch was placed on the mesial and distal surfaces of each tooth to act as a retaining glove for the ligature. The mandibular first molars and third and fourth premolars were ligated with twisted ligatures of 2-0 silk suture and stainless-steel ligature wire for 12 weeks and the maxillary second, third, and fourth premolar were also ligated for 12 weeks to induce the periodontitis. All experiments were performed under anesthesia with Zoletil (15 mg/kg I.M). The ligatures were checked daily and missing ligatures were replaced immediately. During the Periodontal disease induction phase, the dogs were fed a plaque-promoting diet of moistened dog chow. Daily tooth brushing was terminated.

After induction of periodontitis, ligatures were removed, and the animals were assigned to a placebo, positive control (doxycycline), and two treatment groups consisting of five animals each (Table 1). Placebo group was administrated only normal dog chow diet (25 g/kg/day). In doxycycline treatment group, animals were fed normal diet (25 g/kg/day) and doxycycline (5 mg/kg) was orally administrated once per day. The treatment of UP446 was done by feeding the beagle dogs with a regular diet formulated with 0.1% and 0.2% of UP446, determined by weight.

During the treatment period, clinical indices such as plaque index (PI), gingival index (GI), clinical attachment level (CAL), probing pocket depth (PPD) and bleeding on probing (BoP) were measured every two weeks for 12 weeks (Table 2).

### 2.3. Data Analysis

For the statistical analysis, Intergroup comparisons were made by one-way ANOVA, and Turkey test was used as a post-hoc test (*p* < 0.05). All data analyses were performed with SPSS^®^ WIN 12.0.

## 3. Results

### 3.1. Plaque Index (PI)

**Figure 2:**
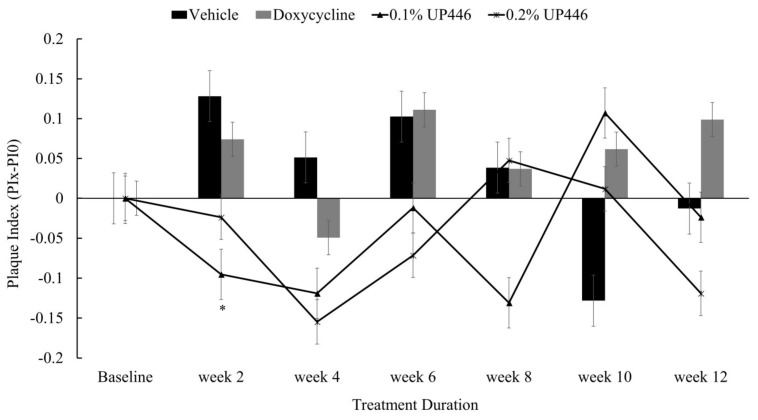
Changes in mean plaque indices in beagle dogs with ligature-induced periodontitis. Values are represented as differences between before and after treatment. *; denote significance at *p* < 0.05 when compared with placebo and treated groups at the same week.

Changes in mean plaque indices in beagle dogs with ligature-induced periodontitis has been shown in Figure 2. Following a 12-week daily treatment, significant differences were not detected between the groups though it is worth noting that for the first six weeks for the 0.2% and eight weeks for the 0.1% UP446 treated dogs showed a reduced trend in plaque formation.

### 3.2. Gingivitis Index (GI)

**Figure 3:**
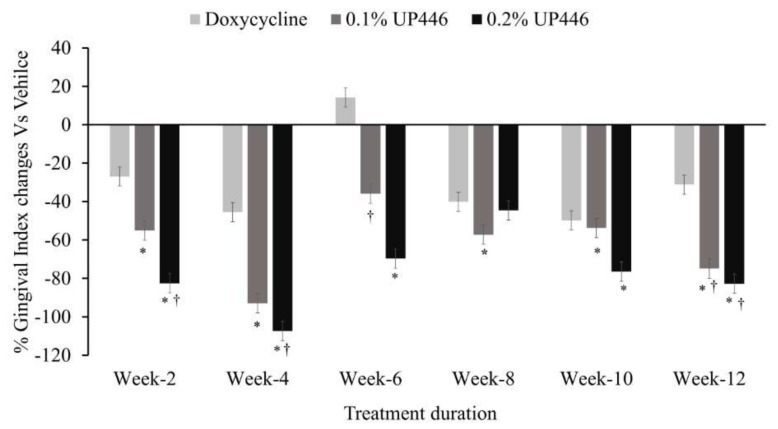
Changes in mean gingival indices in beagle dogs with ligature-induced periodontitis. Values are represented as differences between before and after treatment. *; denote significance at *p* < 0.05 when compared with placebo and treated groups at the same week. †; denote significance at *p* < 0.05 when compared with doxycycline and UP446 groups at the same week.

Changes in mean gingival indices were observed between groups. As seen in Figure 3, dogs treated with either percentage of UP446 showed statistically significant reductions in gingival indices for the duration of study compared to untreated diseased animals. When UP446 was formulated at 0.2% in the diet, the reductions in gingival indices were higher than to that of doxycycline treated dogs. There were ranges of 26.9–49.8%, 55.1–92.9%, and 44.6–107.4% reductions in gingivitis were observed for the dogs treated with Doxycycline, UP446 at 0.1% and UP446 at 0.2%, respectively. At week six, dogs in the doxycycline group showed a 14.2% increase in gingivitis compared to the vehicle treated group.

### 3.3. Probing Pocket Depth (PPD)

**Figure 4:**
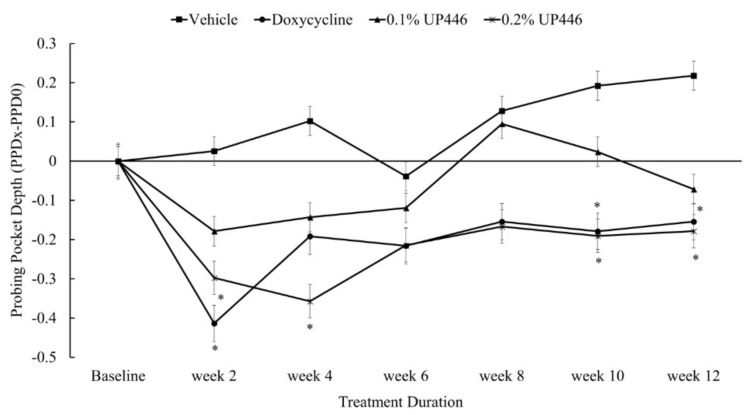
Changes in mean periodontal pocket depth in beagle dogs with ligature-induced periodontitis. Values are represented as differences between before and after treatment. *; denote significance at *p* < 0.05 when compared with placebo and treated groups at the same week.

Following two weeks of oral treatment, there was statistically significant difference in changes in mean periodontal pocket depth in beagle dogs with ligature-induced periodontitis following treatments with doxycycline and 0.2% UP446. At week four, only beagle dogs treated with 0.2% UP446 showed statistically significant reduction in PPD. Treatment groups, doxycycline and 0.2% UP446, showed statistically significant reduction in PPD at weeks 10 and 12. While marked decreases in PPD were observed at weeks six and eight both for the doxycycline and 0.2% UP446 and the changes were not statistically significant. The 0.1% showed statistically non-significant reductions in PPD during the course of treatment (Figure 4). When compared to the vehicle group, there were 16, 7, and 12-fold at week two and 2, 1.4, and 3.5-fold at week four reductions in PPD for the Doxycycline, UP446 at 0.1% and UP446 at 0.2%, respectively. For this parameter, the highest fold decrease in PPD was observed for the doxycycline group at week two.

### 3.4. Clinical Attachment Level Alterations (CAL)

**Figure 5:**
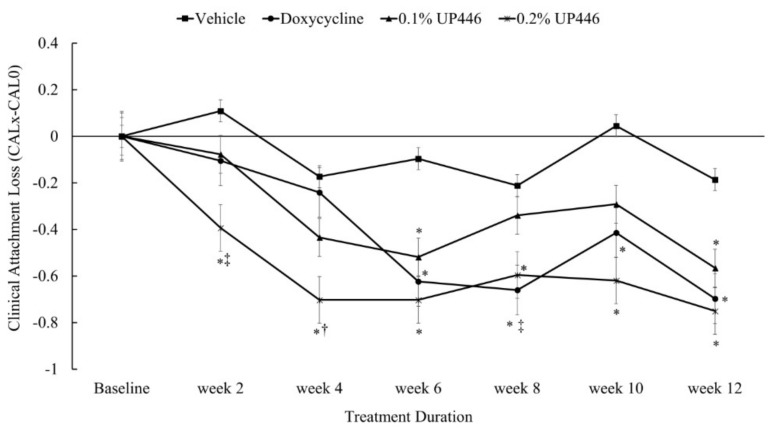
Changes in mean clinical attachment level in beagle dogs with ligature-induced periodontitis. Values are represented as differences between before and after treatment. *; denote significance at *p* < 0.05 when compared with placebo and treated groups at the same week. †; denote significance at *p* < 0.05 when compared with doxycycline and UP446 groups at the same week.

Changes in mean clinical attachment level were statistically significant for beagle dogs treated with 0.2% UP446 as early as two weeks and remained significant for the rest of the duration of study. The doxycycline treated group started the clinical efficacy as of week six and remained significant for the entire study period. The changes observed as a result of treatment with 0.2% UP446 were statistically significant than the reference compound at weeks two and four. UP446 at 0.1% showed statistically significant impact at week 6 and week 12 (Figure 5). We observed a 0.96, 0.71 and 3.61-fold at week two and 1.39, 2.51, and 4.06-fold in CAL were observed for the Doxycycline, UP446 at 0.1% and UP446 at 0.2%, respectively. Here, the highest improvements were observed for the dogs treated with 0.2% UP446.

### 3.5. Bleeding on Probing (BOP)

**Figure 6:**
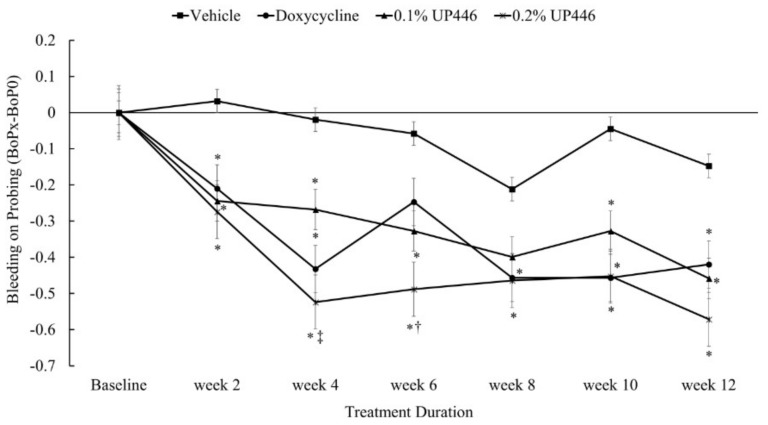
Changes in mean bleeding on probing in beagle dogs with ligature-induced periodontitis. Values are represented as differences between before and after treatment. *; denote significance at *p* < 0.05 when compared with placebo and treated groups at the same week. †; denote significance at *p* < 0.05 when compared with doxycycline and UP446 groups at the same week.

Here, again, changes in mean bleeding on probing were statistically significant for beagle dogs treated with 0.2% UP446 as early as two weeks and remained significant for the rest of the duration of study. Similar efficacies were observed for the doxycycline and the 0.1% UP446 group except at week-6 (for the doxycycline) and at week eight (for the 0.1% UP446) where the reduction in BOP was not statistically significant for this group. Compared to the doxycycline group, beagle dogs treated with 0.2% UP446 showed greater protection in gum bleeding at weeks four and six (Figure 6). There were ranges of 2.8–22.5, 1.9–13.9, and 2.2–27.2-fold reductions in BOP were observed for the dogs treated with Doxycycline, UP446 at 0.1% and UP446 at 0.2%, respectively. Here, again, the highest reductions in BOP were observed for dogs supplemented with UP446 at 0.2%.

## 4. Discussions

In the present study, the use of UP446 in oral care was evaluated at two specific concentrations administered to ligature induced periodontal disease in beagle dogs. Despite the benefit of in vitro assays to study physiological processes that occur during the pathogenesis of periodontitis, the complex host response primarily responsible for this disease cannot be addressed in these assays.

Periodontal disease is the most widespread oral disease in dogs with significant impact on the general wellbeing of the companion animals. It’s been reported that the accumulation of plaques on the tooth surface ultimately causes periodontal disease where the plaque biofilm activates the host inflammatory response, which causes the ligaments and bones that support the tooth to become inflamed and progressively destroyed [22]. Hence, acknowledging inflammation and microbial infection as the core cause for periodontal disease, a composition comprised of the extracts of medicinal plants with known anti-inflammatory and anti-microbial activities could provide the best intervention for the disease. UP446, a botanical composition which consists primarily of baicalin from the root of *Scutellaria baicalensis* Georgi and catechin from the heartwood of *Acacia catechu*, possesses in vitro activities with potential benefits in periodontal disease such as inhibition of COX and LOX enzymes [23] and down regulation of COX-2, TNF-α, IL-1β, IL-6, and NF-κB gene expression [24]. The inflammatory mediators, such as interleukin (IL)-1 and prostaglandin E 2 (PGE 2) play significant roles in the induction of RANKL expression with an ultimate outcome of alveolar bone loss [18]. The extent of COX-2 in the role of periodontal disease has also been reported in association with increased level of PGE2 leading to periodontal tissue destruction. We believe that UP446 has practical application in periodontist as an anti-inflammatory agent where the major proinflammatory cytokines involved in periodontist have been inhibited by the composition at the enzymatic or gene expression level. In fact, UP446 was discovered in our laboratory from random screening of plant extracts for COX and LOX dual modulation where these enzymes have been identified as a key player in gingivitis [13,25,26,27]. Recently, in the ligature induced murine model, *Scutellaria baicalensis* extract showed protection in the destruction of periodontal ligament by inhibiting the production of IL-1β, IL-6, IL-8, and TNF-α cytokine mRNA expression in gingival tissues [28]. Unchecked, these cytokines could have been acted in consortium to initiate and sustain inflammatory response in periodontitis which could ultimately lead to fully developed periodontal disease.

Moreover, significant body of evidence described the effect of baicalin and catechin on the predominant periodontopathogens responsible for instigating periodontal disease. The development of gingivitis is accompanied by substantial increases in the number of Gram-negative anaerobic rods such as *Porphyromonas gingivalis* and *Prevotella* spp., which have been strongly implicated in periodontitis. The major active compounds of UP446 have been reported with bactericidal activities on these pathogens in various preparations. For example, Catechin from green tea showed a bactericidal effect against the principal oral pathogen *Porphyromonas gingivalis* and *Prevotella* spp. in vitro with an MIC of 1.0 mg/mL [29]. The authors also reported that when strips containing green tea catechin used as a slow release for local delivery system, there was a marked decrease in pocket depth and peptidase activities in the gingival fluid compared to the placebo group following 8 weeks of treatment [29]. Other studies have also reported similar outcomes in that a green tea preparation that consists mainly of catechin inhibits the growth of *Streptococcus mutans* and *Lactobacillus* spp. (principal bacteria for dental caries) [30] and the adherence *P. gingivalis* to oral epithelial cells at concentrations below 0.25 mg/mL [31]. The significant outcome observed in our study, in part, could be as a result of catechin.

Similarly, extract from *Scutellaria baicalensis* has shown activities on obligate biofilm organisms such as *Streptococcus mutans* which is commonly identified as the principal etiological agent in dental carries and these extracts were found to suppress these pathogens and were suggested for the prevention of dental carries [32]. In a similar study, when the dried root extract of *Scutellaria baicalensis* was tested on eleven commonly isolated periodontopathogens, a concentration of 2% and 3.16% were found as the bacteriostatic and bacteriocidal concentrations for these pathogens suggesting the effective use of this extract in oral care [33]. The antimicrobial activity of baicalin has also been demonstrated by its inhibitory effect on collagenase activity of *P. gingivalis*, which is an important effector in the onset and the establishment of periodontal disease [16]. Arweiler et al. have examined the potential use of *Scutellaria baicalensis* for oral care by using experimentally induced gingivitis in humans. Following gingivitis induction, subjects were treated with the extract of *Scutellaria baicalensis* formulated tooth paste slurry at 0.5% for 1 min in comparison with placebo group where gingival index (GI), plaque index (PI), and biofilm vitality (VF%) were assessed at days 0, 14, and 21. While statistically significant improvements were observed for the PI and GI on days 14 and 21 compared to baseline, the reductions in VF achieved the significance on day 21. The authors suggest the toothpaste formulation was able to significantly reduce the extent of gingivitis, plaque development, and vital flora in their study [34]. These beneficial activities of *Scutellaria baicalensis* or tis active baicalin reinforces a potentiate efficacy formulated together with catechin against periodontal disease.

The role of reactive oxygen species in periodontal disease have been described in the literature [35]. During periodontal disease, most of the periodontal tissue destruction is caused by an unbalanced host response to the etiologic microbes (predominantly gram negative anaerobic or facultative bacteria within the subgingival biofilm) and their products where the loss in ROS homeostatic balance plays key roles in the process. The significant amelioration of periodontal disease observed in our study could be partially explained by the strong anti-oxidant activity of UP446 or its individual active components, baicalin, and catechin [36,37].

Several criteria have been used to establish the diagnosis of periodontal disease [5]. Among these combinations of plaque/calculus with indices for gingivitis, pocket depth, clinical loss of attachment, and bleeding on probing are widely referenced in clinical settings. We used these parameters in our study to monitor disease model induction and efficacy of intervention. With the exception of plaque index (which showed strong trend of reduction), administration of UP446 formulated diet to beagle dogs for 12 weeks at 0.1% and 0.2% resulted in statistically significant reductions in gingivitis index, pocket depth, loss of attachment and gum bleeding. In some parameters such as gingivitis index, attachment loss and gum bleeding, treatment with UP446 at 0.2% resulted better efficacy than the positive control doxycycline in varieties of time points. These findings are in parallel with activities that have been described above for the individual active components, baicalin and catechin from *Scutellaria baicalensis* and *Acacia catechu* extract. Therefore, UP446, a botanical composition which consists primarily of baicalin from *Scutellaria baicalensis* Georgi and catechin from *Acacia catechu* could potentially be used for oral care due to its antimicrobial activity against periodontal bacteria, strong anti-inflammatory, and anti-oxidant activities.

While data compiled in this study have substantial implications suggesting the use of UP446 for oral care, the study has some limitations. We did not determine oral vital flora, and blood biomarkers which may have addressed some of the mechanisms of actions of test compounds. As indicated by the plaque index, which may have been impacted more by mechanical exposure than biochemical effect of test compounds, delivery mechanism of test compounds could have a better outcome if they were formulated in dog treats like chew-bone. These preparations will engage the animals to chew longer and hence give the compounds more resident time in the oral cavity.

## 5. Conclusions

Collectively, the active constituents of UP446 have an in vitro antibacterial effect against periodontopathic bacteria and are also known to inhibit destruction of the periodontal tissue initiated by inflammation in association with excessive ROS generation. UP446 significantly improved clinical parameters indicative of periodontal disease. Due to marked similarities in anatomical and physiological features between dogs and human periodontal disease, data generated in this study could possibly be used to justify the use of UP446 both in the companion animals and human oral care as an adjunctive therapy.

## Figures and Tables

**Figure 1:**
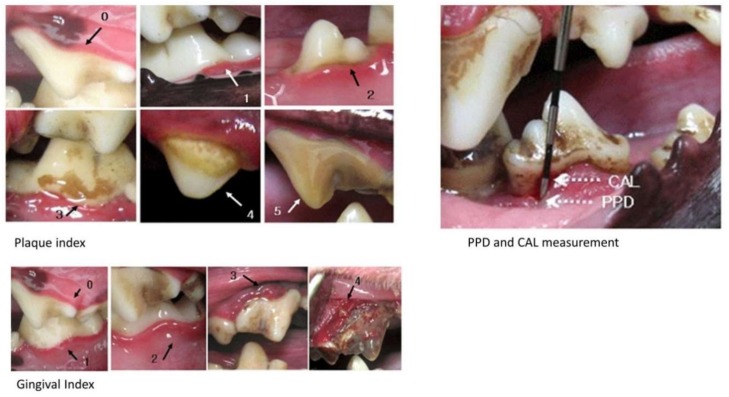
Clinical parameters for gingivitis. Arrows indicate the corresponding grades for Plaque index, and Gingival index as defined in the Table 2. PPD: probing pocket depth and CAL: clinical attachment level.

**Table 1:** Dosing of each treatment.

Group	Treatment	Dosage	Duration of Treatment (Weeks)	No. of Animal (Male: Female)	Amount of Diet (g/kg/day)	Route of Treatment
I	Placebo	0	12	5 (1:4)	25	-
II	Doxycycline	5 mg/kg	12	5 (1:4)	25	p.o.q.d
III	UP446	0.1% in diet	12	5 (2:3)	25	Formulated in Diet
IV	UP446	0.2% in diet	12	5 (2:3)	25	Formulated in Diet

**Table 2:** Clinical parameters (Figure 1).

Parameters	Grades	
PI ^1^	0	No Plaque
	1	A film of plaque adhering to the free gingival margin and adjacent area of the tooth. (not more than 1 mm)
	2	Moderate accumulation of soft deposits within the gingival pocket, or on the tooth and gingival margin which can be seen with the naked eye. (less than one half of crown)
	3	Abundance of soft matter within the gingival pocket and/or on the tooth and gingival margin. (more than one half of crown)
GI ^2^	0	Absence of inflammation
	1	Mild inflammation—slight change in color of gingival margin and little change in texture
	2	Moderate inflammation—moderate glazing, redness, edema, and hypertrophy. Bleeding on pressure.
	3	Severe inflammation—marked redness and hypertrophy, spontaneous bleeding and ulceration
PPD ^3^	The distance between the gingival margin and the bottom of the probable pocket	
CAL ^3^	The distance between the cement enamel junction and the bottom of the probable pocket	
BoP ^3^	0	Absence of bleeding within 10 s following probing
	1	Presence of bleeding within 10 s following probing

^1^ Silness J, Loe H. Periodontal disease in pregnancy. II. Correlation between oral hygiene and periodontal condition. Acta Odontol Scand 1964; 22: 121–135. ^2^ Loe H, Silness J. Periodontal disease in pregnancy. I. Prevalence and severity. Acta Odontol Scand 1963; 21: 533–551. ^3^ Wennstrom JL, Newman HN, MacNeill SR, et al. Utilization of locally delivered doxycycline in non-surgical treatment of chronic periodontitis. A comparative multi-centre trial of 2 treatment approaches. J Clin Periodontol 2001; 28: 753–761.
